# A Rare Case of Chronic Thromboembolic Pulmonary Hypertension in an Elderly With Atrial Septal Defect and Eisenmenger Syndrome

**DOI:** 10.7759/cureus.90281

**Published:** 2025-08-17

**Authors:** Ernestine Faye S Tan, Sakar B Gharti, Danielle Grace S Tan

**Affiliations:** 1 Internal Medicine, Interfaith Medical Center, New York, USA; 2 General Practice, University of Santo Tomas Faculty of Medicine and Surgery, Manila, PHL

**Keywords:** arrhythmia, atrial flutter, atrial septal defect, cardiology, chronic thromboembolic pulmonary hypertension (cteph), congenital heart disease, eisenmenger syndrome, heart failure, pulmonary hypertension, pulmonary thromboembolism

## Abstract

Atrial septal defects (ASDs) are among the most common congenital heart anomalies and often remain undetected until adulthood. While small ASDs may close spontaneously, larger unrepaired defects can lead to serious complications such as right heart failure, pulmonary arterial hypertension, Eisenmenger syndrome (ES), and thromboembolic events.

We present a case of a 66-year-old Filipino female with a longstanding unrepaired secundum-type ASD, who was admitted with progressive dyspnea and hypoxemia. The patient developed atrial flutter, and further workup revealed severe pulmonary hypertension, right ventricular failure, and pulmonary embolism. Imaging confirmed chronic thromboembolic pulmonary hypertension (CTEPH), a rare but life-threatening complication in patients with ES. Despite aggressive medical management, including anticoagulation, antibiotics for pneumonia, and antiarrhythmic therapy, the patient’s condition deteriorated, and she eventually succumbed to her illness.

This case highlights the critical importance of early detection and surgical repair of large ASDs to prevent irreversible pulmonary vascular disease and associated complications such as ES and CTEPH, particularly in resource-limited settings where definitive interventions are often inaccessible.

## Introduction

Congenital heart disease (CHD) is the most common category of birth defects, accounting for nearly one-third of all major congenital anomalies [1,2]. Atrial septal defect (ASD) is one of the most prevalent types of CHD, with a birth prevalence of approximately 2.5 per 1,000 live births, and 25-30% of these cases are diagnosed in adulthood [1,3].

ASD is characterized by a defect in the interatrial septum that creates a left-to-right shunt, allowing oxygenated blood from the left atrium to flow into the right atrium [1]. Because systemic oxygenation is initially preserved, ASDs are classified as acyanotic CHDs [1,2]. However, if left unrepaired, a large ASD can result in right heart volume overload, pulmonary hypertension, and eventually Eisenmenger syndrome (ES).

Eisenmenger syndrome develops when increased pulmonary blood flow induces irreversible pulmonary vascular remodeling and elevated pulmonary arterial pressure [4,5]. Once right-sided pressure exceeds left-sided pressure, the shunt reverses to right to left, resulting in systemic hypoxemia and cyanosis [4]. This progression is observed in approximately 50% of unrepaired large ventricular septal defects (VSDs) and patent ductus arteriosus (PDA), but in only 10% of unrepaired ASDs [5].

Pulmonary thromboembolism (PTE) is a known but rare complication of Eisenmenger syndrome, with a reported incidence of 21-29% [6,7]. The pathogenesis is multifactorial, involving in situ thrombosis due to endothelial dysfunction, vascular remodeling, and impaired fibrinolysis [6-8]. The development of thrombotic complications significantly worsens morbidity and mortality and underscores the importance of early diagnosis and management [7].

ES represents the most advanced and severe form of pulmonary arterial hypertension (PAH) related to CHDs [5]. In a study by Daliento and associates, it was found that patients with ES had earlier clinical deterioration and a shorter survival rate, with the most common cause of mortality being sudden cardiac death (29.5%), followed by heart failure (22.9%) [6]. Of 188 patients, 25 (13.2%) had PTE at a mean age of 35.2 ± 13.4 years [6]. The coexistence of chronic thromboembolic pulmonary hypertension (CTEPH) further exacerbates right heart dysfunction and significantly worsens the clinical trajectory.

This report presents a rare case of an adult patient with a long-standing, unrepaired secundum-type ASD who developed Eisenmenger syndrome complicated by CTEPH and ultimately succumbed to her illness.

## Case presentation

A 66-year-old Filipino female from an economically disadvantaged population in Manila, Philippines, presented with progressive dyspnea that started four days prior to admission. Initially, she could tolerate light activity, but on the day of admission, she became severely dyspneic at rest and could only speak in short phrases.

She had a history of hypertension for over 10 years and was diagnosed with a secundum-type ASD at age 17, identified incidentally during a pre-employment chest radiograph. Surgical closure was advised for a defect of 8 mm, but it was not pursued due to financial constraints. She also had a history of bronchial asthma (on as-needed salbutamol), multinodular toxic goiter (on propylthiouracil), and a prior cerebrovascular accident in 2013 with full recovery and no residual neurologic deficits.

Four days prior to admission, she developed dyspnea on minimal exertion, initially attributed to asthma, but the shortness of breath did not respond to inhalers. Two days later, her symptoms progressed to include orthopnea and dyspnea on speaking short sentences. She began sleeping upright and presented to the emergency department with severe air hunger.

On physical examination, she was cyanotic and tachycardic with an irregularly irregular rhythm. She had distended neck veins, bilateral mid-to-basal crackles, a systolic ejection murmur, a right ventricular heave, and a displaced apex beat. A wide, fixed splitting of S2 was noted.

**Figure 1:**
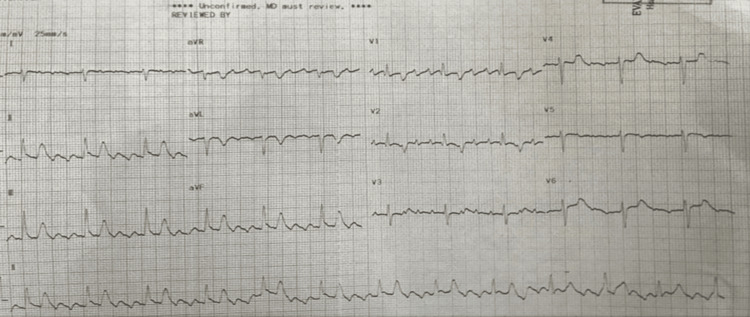
Electrocardiogram on admission shows atrial flutter.

**Figure 2:**
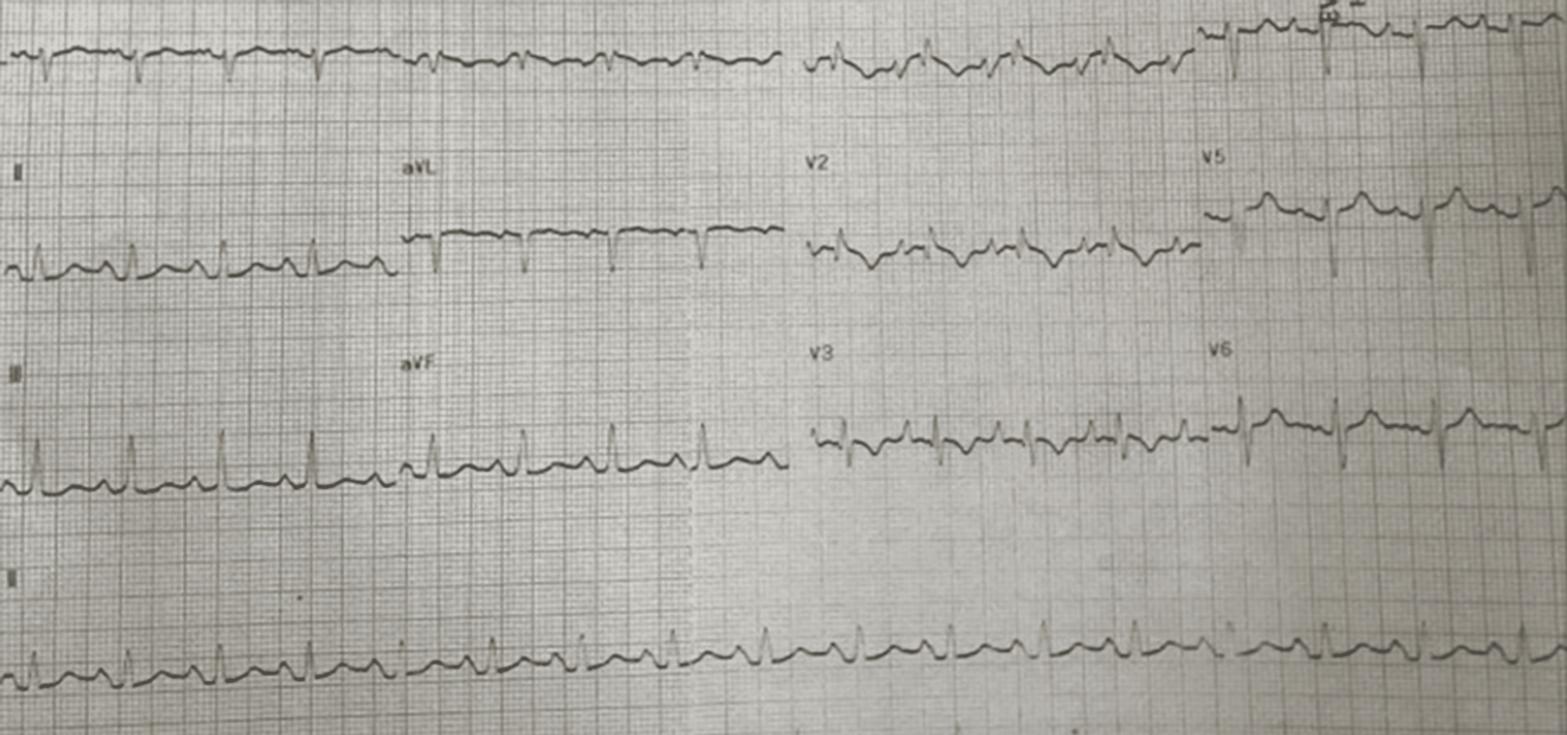
Electrocardiogram after medication administration, which reverted the rhythm back to sinus.

She was hypoxemic, with an oxygen saturation of 78% on a face mask at 10 liters. Her respiratory rate was 35 breaths per minute. She was afebrile but tachycardic at 115 beats per minute. Her blood pressure was 92/57, with a mean arterial pressure of 65. For hypoxemia and severe respiratory distress, she was intubated and placed on mechanical ventilation. Electrocardiogram (EKG) revealed atrial flutter (Figure 1), which converted to sinus rhythm after administration of digoxin and verapamil (Figure 2).

**Figure 3:**
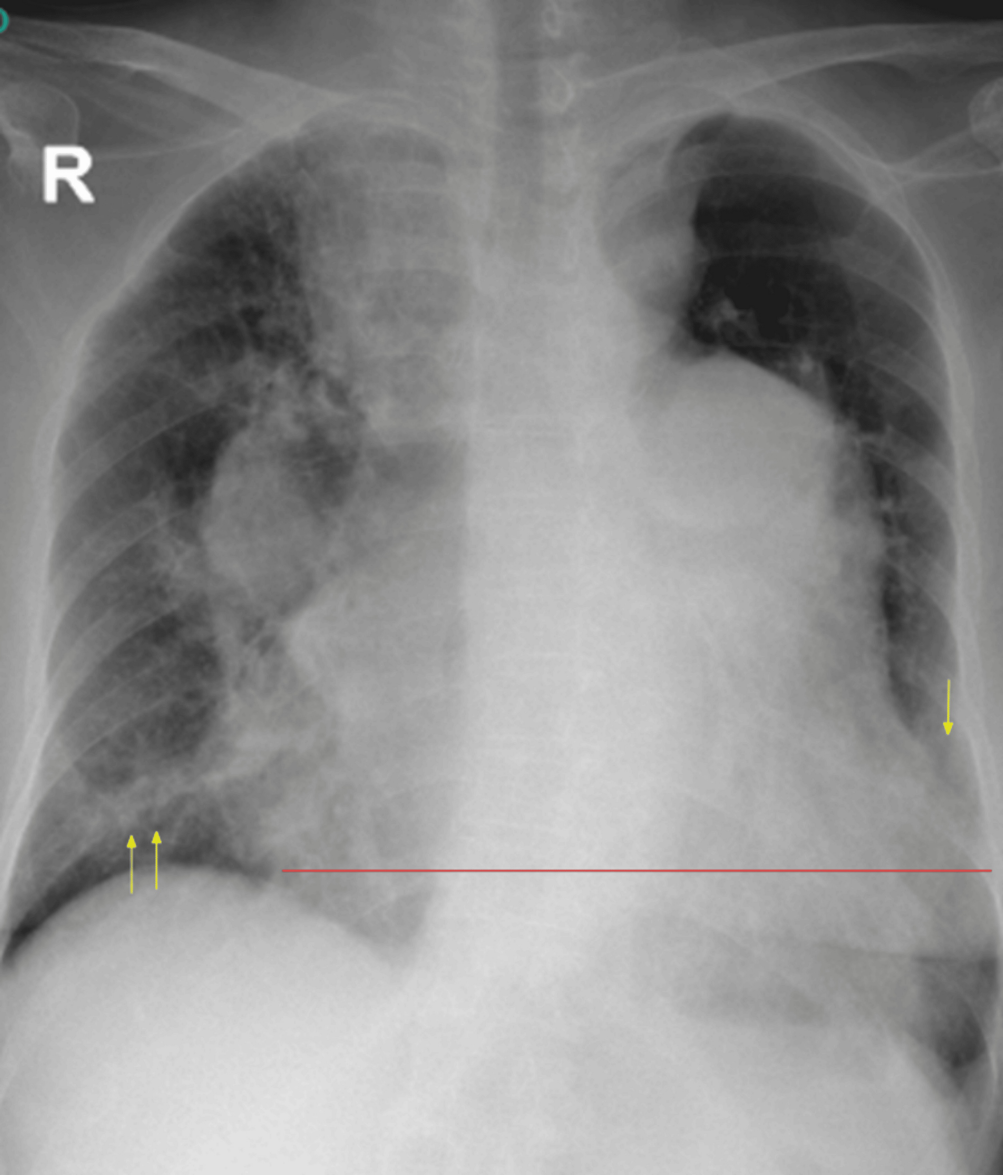
Chest radiograph showing widened superior mediastinum, cardiomegaly (solid red line), and bibasal lung infiltrates (yellow arrows), suggestive of bilateral pneumonia.

Chest radiography showed bibasal pneumonia and cardiomegaly (Figure 3). The patient was started on piperacillin-tazobactam and levofloxacin for high-risk pneumonia.

**Figure 4:**
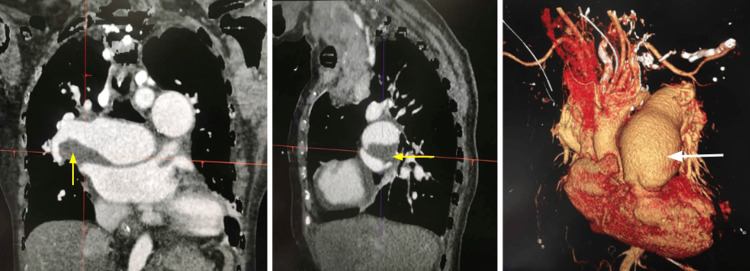
Computed tomography of the chest with contrast revealed pulmonary embolism in the right main and right proximal descending pulmonary arteries (yellow arrows), and a dilated pulmonary artery (white arrow).

Her Wells score was 6, indicating moderate risk for PTE. D-dimer was elevated (1.4 times the upper limit of normal), prompting anticoagulation with enoxaparin. A computed tomography (CT) of the chest was done, which showed an increase in dilatation of the main pulmonary trunk, right and left main pulmonary arteries, and descending interlobar artery. Acute and chronic pulmonary thromboembolism of the right main and proximal descending pulmonary arteries were seen (Figure 4). There was relative oligemia and lucency of the right middle lobe. Additionally, cardiomegaly and bilateral pneumonia were also seen, along with the patient’s multinodular goiter with intrathoracic extension. Surgical thromboendarterectomy was discussed with the family, who opted not to proceed with the procedure due to its high-risk nature.

Venous duplex showed no deep vein thrombosis. Meanwhile, echocardiography revealed an ejection fraction of 67%, a large secundum ASD with Qp/Qs of <1, dilated right atrium and ventricle, severe pulmonary hypertension, tricuspid and pulmonic regurgitation, and small pericardial effusion.

On the 13th hospital day, the patient experienced recurrent atrial flutter, necessitating an amiodarone drip. Her pneumonia progressed despite antibiotics. Piperacillin-tazobactam was escalated to meropenem. Endotracheal aspirate revealed moderate growth of *Elizabethkingia meningoseptica*, hence ciprofloxacin was started based on sensitivity results. Blood cultures revealed *Acinetobacter baumannii* growth, hence gentamicin was started as well.

Serial arterial blood gases showed persistent worsening hypoxemia and hypercapnia, consistent with Eisenmenger physiology. On the 16th hospital day, the patient developed chest pain and worsening dyspnea despite being intubated. Troponin I was markedly elevated (21 times the upper limit of normal), and she was treated for non-ST elevation myocardial infarction (NSTEMI). Despite medical management, her condition deteriorated. The family opted for no further resuscitative measures, and the patient expired shortly after a fatal arrhythmia later that day.

## Discussion

ASD is the second most common congenital cardiac defect, following VSD [8]. Approximately 75-80% of ASDs are of the secundum type [8]. According to a study by Radzik et al., ASDs measuring less than 3 mm have a 100% likelihood of spontaneous closure by 18 months of age. Defects measuring 3-5 mm should be re-evaluated by one year of age, as a significant proportion will close spontaneously [9]. Similarly, 5-8 mm defects should be re-evaluated by 15 months, with more than 80% closing without intervention [9]. In contrast, ASDs larger than 8 mm rarely close spontaneously, and surgical repair should be considered [9]. In our case, the patient was diagnosed with an 8 mm ASD during adolescence, significantly reducing the likelihood of spontaneous closure and highlighting a missed opportunity for early surgical intervention to prevent complications.

ASDs often remain undiagnosed well into adulthood due to their asymptomatic nature during childhood. However, some patients may present with non-specific symptoms such as easy fatigability, recurrent respiratory infections, palpitations, or exertional dyspnea [10]. More serious complications, including arrhythmias, syncope, stroke, and heart failure, can also occur [10]. Consistent with these findings, our patient had suffered a cerebrovascular accident a decade prior to presentation.

On physical examination, typical findings may include a hyperdynamic right ventricular impulse, palpable pulmonary artery pulsation, an ejection click, wide fixed splitting of the second heart sound (S2), and systolic ejection or mid-diastolic murmurs [10]. Transthoracic echocardiography remains the most widely used and cost-effective modality for diagnosing ASD. When Eisenmenger physiology or a right-to-left shunt is suspected, contrast echocardiography may aid in confirming the diagnosis [10,11].

In this patient, the uncorrected ASD led to progressive pulmonary vascular remodeling and severe pulmonary arterial hypertension, ultimately culminating in Eisenmenger syndrome. This condition is characterized by right-to-left shunting, systemic hypoxemia, and multi-organ dysfunction. Unfortunately, further imaging, such as repeat echocardiography or ventilation-perfusion scanning, was not pursued due to financial limitations. While arterial blood gas (ABG) analysis can reflect the degree of shunting, it is a suboptimal method for monitoring disease progression. In this case, persistent desaturation and hypercapnia despite ventilatory support indicated irreversible disease.

Large, unrepaired ASDs can lead to a spectrum of complications, including right-sided heart failure, recurrent pulmonary infections, pulmonary arterial hypertension, atrial arrhythmias, paradoxical emboli, and Eisenmenger syndrome [9-11]. Our patient experienced all of these sequelae, including severe pneumonia, right heart failure, pulmonary hypertension, atrial flutter and fibrillation, acute pulmonary embolism, CTEPH, and a prior stroke. EKG signs such as a right bundle branch block (RBBB) and right axis deviation, particularly with symptoms of dyspnea and hypoxemia, can suggest right-sided volume overload or pulmonary hypertension. Findings should prompt further investigations of causes, such as underlying congenital heart diseases and the development of Eisenmenger syndrome.

CTEPH is a rare but severe complication of ASD. It arises from unresolved or recurrent pulmonary emboli and is more likely in individuals with pre-existing pulmonary vascular disease [11,12]. Proposed mechanisms include in situ thrombosis driven by turbulent flow, endothelial dysfunction, and a hypercoagulable state [11,12]. According to Broberg et al., 21-29% of Eisenmenger syndrome patients have a significant risk of pulmonary artery thrombus formation [13]. CTEPH, defined as chronic thromboembolic occlusion of the pulmonary vasculature, is best diagnosed by computed tomography pulmonary angiography (CTPA) or a ventilation-perfusion scan [11,12]. Data from the International CTEPH Registry indicate that 75% of affected patients have a history of acute pulmonary embolism [14], with the post-embolism incidence of CTEPH estimated at 0.4-6.2% [15].

The only curative treatment for CTEPH is pulmonary thromboendarterectomy, which involves the surgical removal of thromboembolic material from the pulmonary arteries [12]. However, many patients are ineligible for surgery due to advanced disease or comorbidities. Riociguat, a soluble guanylate cyclase stimulator, has been approved for inoperable CTEPH, though it offers symptomatic relief rather than a definitive cure [12]. In our patient, end-stage Eisenmenger physiology and poor surgical candidacy limited treatment options to supportive and palliative care.

At present, no pharmacologic therapy has demonstrated the ability to reverse Eisenmenger syndrome, improve survival, or reduce morbidity conclusively. Selective pulmonary vasodilators such as bosentan and sildenafil have shown benefit in pulmonary arterial hypertension, but their use in Eisenmenger patients remains controversial due to limited patient populations and a lack of randomized controlled trials. In resource-constrained settings, treatment is largely symptomatic and palliative. Surgical options such as heart-lung transplantation or lung transplantation with repair of the cardiac defect offer definitive therapy but carry significant perioperative morbidity and mortality.

Preventive strategies remain paramount. Early detection and timely surgical repair of congenital cardiac defects are the most effective measures to prevent the progression to Eisenmenger syndrome and its associated complications.

## Conclusions

This case highlights the serious consequences of an untreated ASD, which can progress to Eisenmenger syndrome and CTEPH. Once pulmonary vascular disease becomes irreversible, management becomes limited, and the focus shifts to palliative care. Early detection and surgical closure of large ASDs are vital to prevent complications such as pulmonary hypertension, arrhythmias, stroke, infections, and thromboembolism. Unfortunately, in resource-limited settings where diagnostic and surgical services are scarce, patients are at high risk of disease progression.

Once ES develops, management focuses on symptom control, often relying on imaging tools like echocardiography to monitor progression. However, in areas lacking such resources, clinicians must rely on clinical signs and worsening blood gas measurements. Medications such as bosentan and sildenafil may provide symptom relief, though their impact on survival remains uncertain. While heart-lung transplantation is the only curative option for advanced ES, it is rarely feasible due to cost and complexity. This case highlights the importance of early intervention, vigilant follow-up, and public health strategies aimed at improving access to congenital cardiac care.
